# Newborn with massive right eye proptosis revealing quintessence cystic orbital teratoma

**DOI:** 10.11604/pamj.2021.39.110.30023

**Published:** 2021-06-07

**Authors:** Rohan Kumar Singh, Gaurav Vedprakash Mishra

**Affiliations:** 1 Department of Radiodiagnosis, Jawaharlal Nehru Medical College, Datta Meghe Institute of Medical Sciences, Sawangi (Meghe), Wardha, India

**Keywords:** Orbital teratoma, ultrasonography, orbital mass, neonate

## Image in medicine

A full-term 2-day-old female neonate weighing about 2.17 kilogram, born by normal delivery at 37 weeks of gestation was referred to the ophthalmology with a massive mass of the right eye. On clinical examination, there was severe proptosis of the right eye with a positive transillumination test with normal left eye. The orbital mass was hard in consistency with few soft areas and it was covering the right midface. The eyeball was pushed on anteroinferior side with severe conjunctival chemosis and corneal erosion due to exposure to keratopathy with absent papillary reflex. For further routine investigation the baby was referred to the Department of Radiology for a B-scan ultrasound. On ultrasound, multiple anechoic cystic areas were seen anterior to globe pushing it anteroinferior, the hetero-echoic lesion was seen filling right eyeball and lesion was involving ocular muscles.

**Figure 1:**
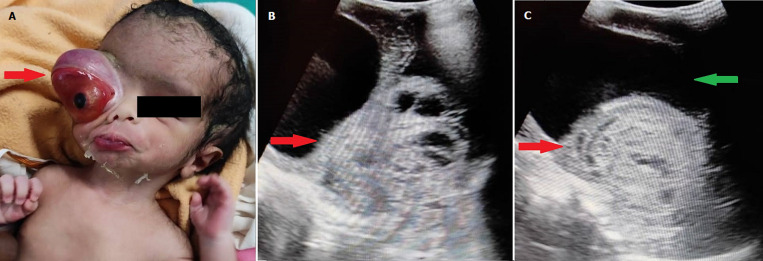
A) severe right eye proptosis with eyeball pushed out (red arrow); heterogeneous lesion with multiple anechoic cystic areas (red arrow); B) ultrasound images; C) heterogeneous well-defined lesion (red arrow), globe is pushed anteriorly (green arrow)

